# Multiple myeloma presenting as an intracranial plasmacytoma: a case report

**DOI:** 10.1186/1757-1626-2-9110

**Published:** 2009-11-30

**Authors:** Tadashi Terada

**Affiliations:** 1 Department of Pathology, Shizuoka City Shimizu Hospital, Miyakami 1231, Shimizu-Ku, Shizuoka, 424-8636, Japan

## Abstract

Multiple myeloma presenting as an intracranial tumor (plasmacytoma) is very rare. An 81-year-old woman was admitted to our hospital because of gait disturbance. A blood laboratory test revealed a mildly increased lactate dehydrogenase (236 IU/L) and glucose (121 mg/dl). Blood protein fractions were normal. Brain computed tomography and magnetic resonance imaging revealed an intracranial mass (5 × 4 × 3 cm) in the brain base next to the clavus, and it was clinically diagnosed as chordoma. An excision of the brain tumor was performed. Imaging modalities including ultrasound, x-ray, computed tomography, magnetic resonance imaging and positron emission tomography did not reveal any tumors other than the brain tumor. The tumor was soft, fragile, and bloody. Microscopically, a monotonous proliferation of atypical plasma cells with hyperchromatic nuclei was recognized. Histochemically, the tumor cells were pyroninophilic and the congo-red stain revealed amyloidosis. Immunohistochemically, the tumor cells were positive for κ-chain and negative for cytokeratin, epithelial membrane antigen, vimentin, CD45, CD20, CD45RO, λ-chain, IgM, IgA, IgG, synaptophysin, chromogranin, S100 protein, desmin, α-smooth muscle antigen, myoglobin, p53 protein, and glial fibrillary acidic protein. The Ki-67 labeling was 11%. Intracranial plasmacytoma was pathologically diagnosed. The patient was treated by adjuvant chemoradiation, and entered into the complete remission stage. However, multiple metastases emerged in the vertebral bones and ribs six months after the remission. A diagnosis of multiple myeloma was made. The urine revealed Bence-Jones protein of monoclonal IgG κ-chain type, but blood M protein was not recognized. The patient's condition gradually deteriorated. The patient died of respiratory failure due to bronchopneumonia 18 months after the admission. The present case indicates that multiple myeloma may manifest as an intracranial brain tumor (plasmacytoma).

## Introduction

Multiple myeloma (MM) is a serous malignant neoplasm of bone marrow, and mostly occurs in the elderly persons. The tumor cells of this disease are plasma cells, and produce immunoglobulins and/or light chains. It is occasionally complicated by amyloidosis. MM is usually detected in bones with characteristic features of punched-out lesions. MM presenting as an intracranial brain tumor or intracranial plasmacytoma is very rare; a survey of the world literature revealed only 7 such cases [1-7]. Here, the author reports an intracranial brain tumor of plasmacytoma, which later developed into MM.

## Case presentation

**Figure 1:**
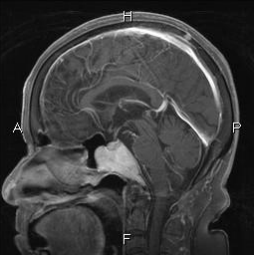
**MRI findings**. An intracranial tumor is present in the brain base next to the clavus.

An 81-year-old woman complained of gait disturbance, and consulted to our hospital for scrutiny. A blood laboratory test revealed a mildly elevated LDH (236 IU/L) and glucose (121 mg/dl). Blood protein fractions were normal, and no hyper-λ-globurinemia and M-protein were recognized. Brain X-P, CT and MRI revealed an intracranial mass (5 × 4 × 3 cm) in the brain base next to the clavus (Figure 1), and it was clinically diagnosed as chordoma by radiologists and neurosurgeons. An excision of the brain tumor was performed. Imaging modalities including US, X-P, CT, MRI and PET revealed no tumors in the extracranial locations.

**Figure 2:**
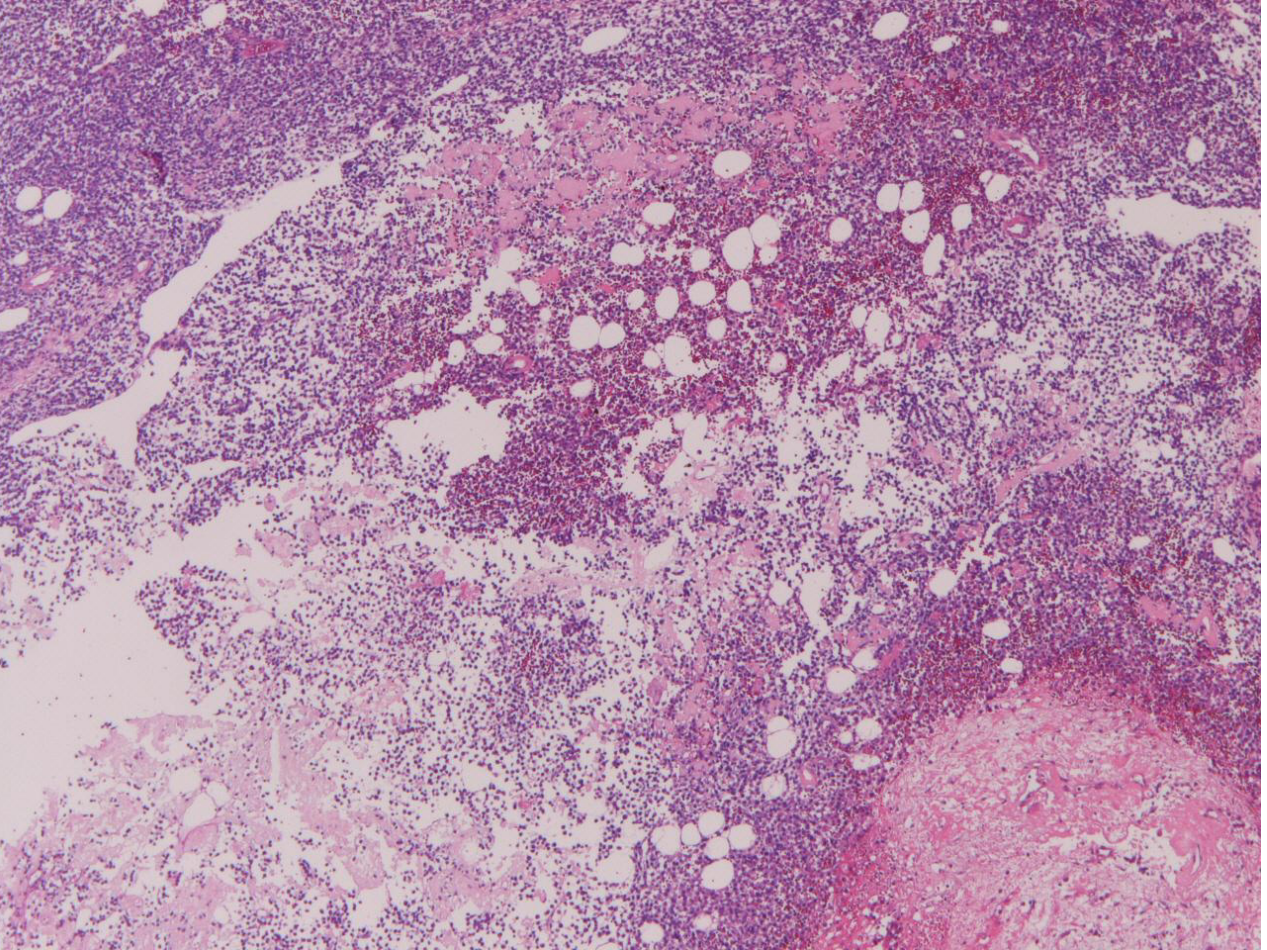
**Low power microscopic view**. Proliferation of small atypical cells is seen. HE, ×10.

**Figure 3:**
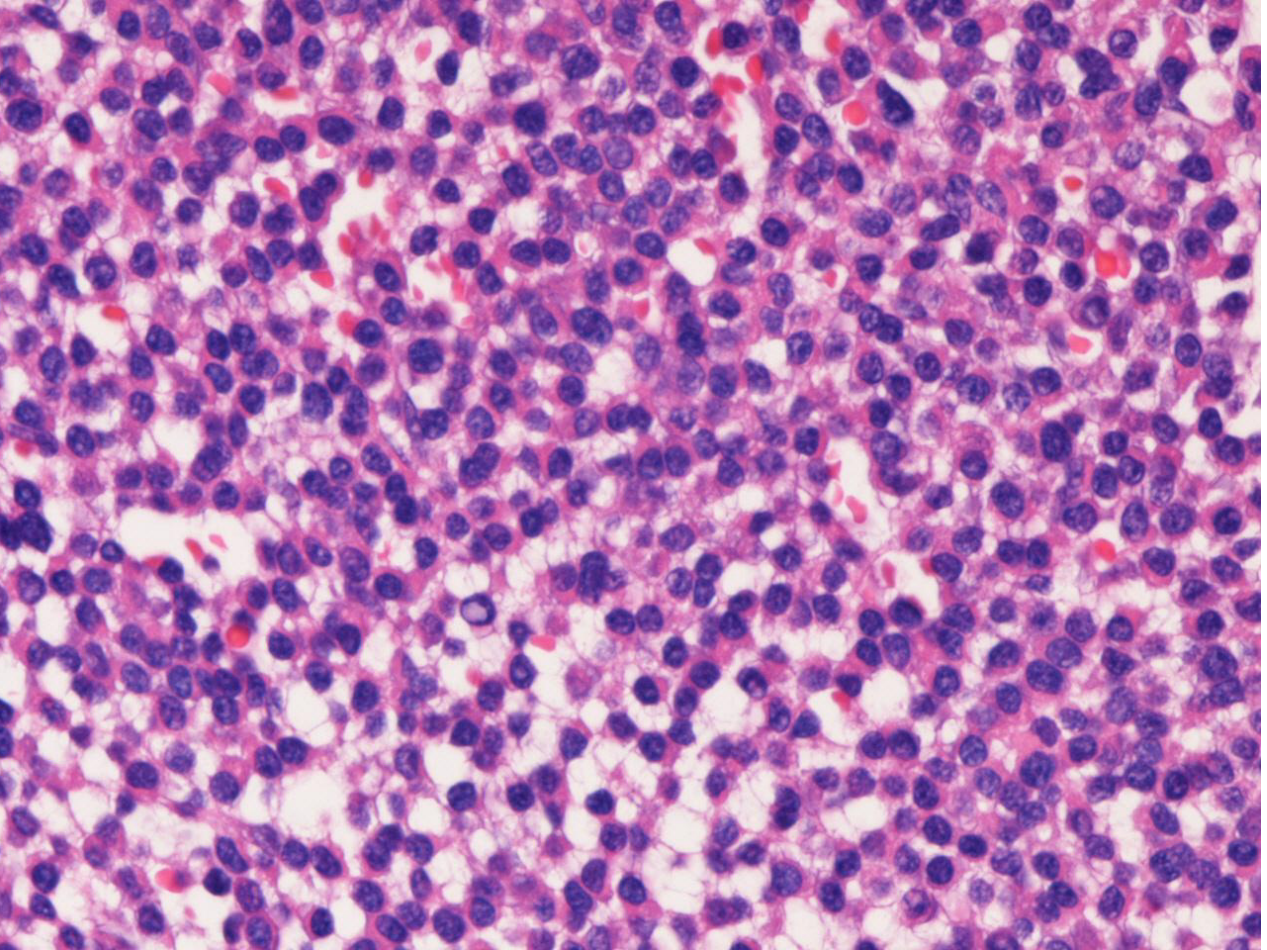
**Higher power microscopic view**. The tumor cells are round cells with eccentrically located nuclei. The nuclei show hyperchromasia. HE, ×400.

**Figure 4:**
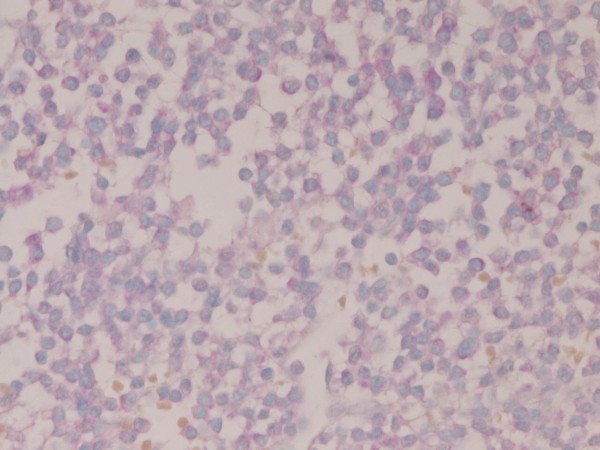
**The tumor cells are pyroninophilic**. Methylgreen pyronine, ×400.

**Figure 5:**
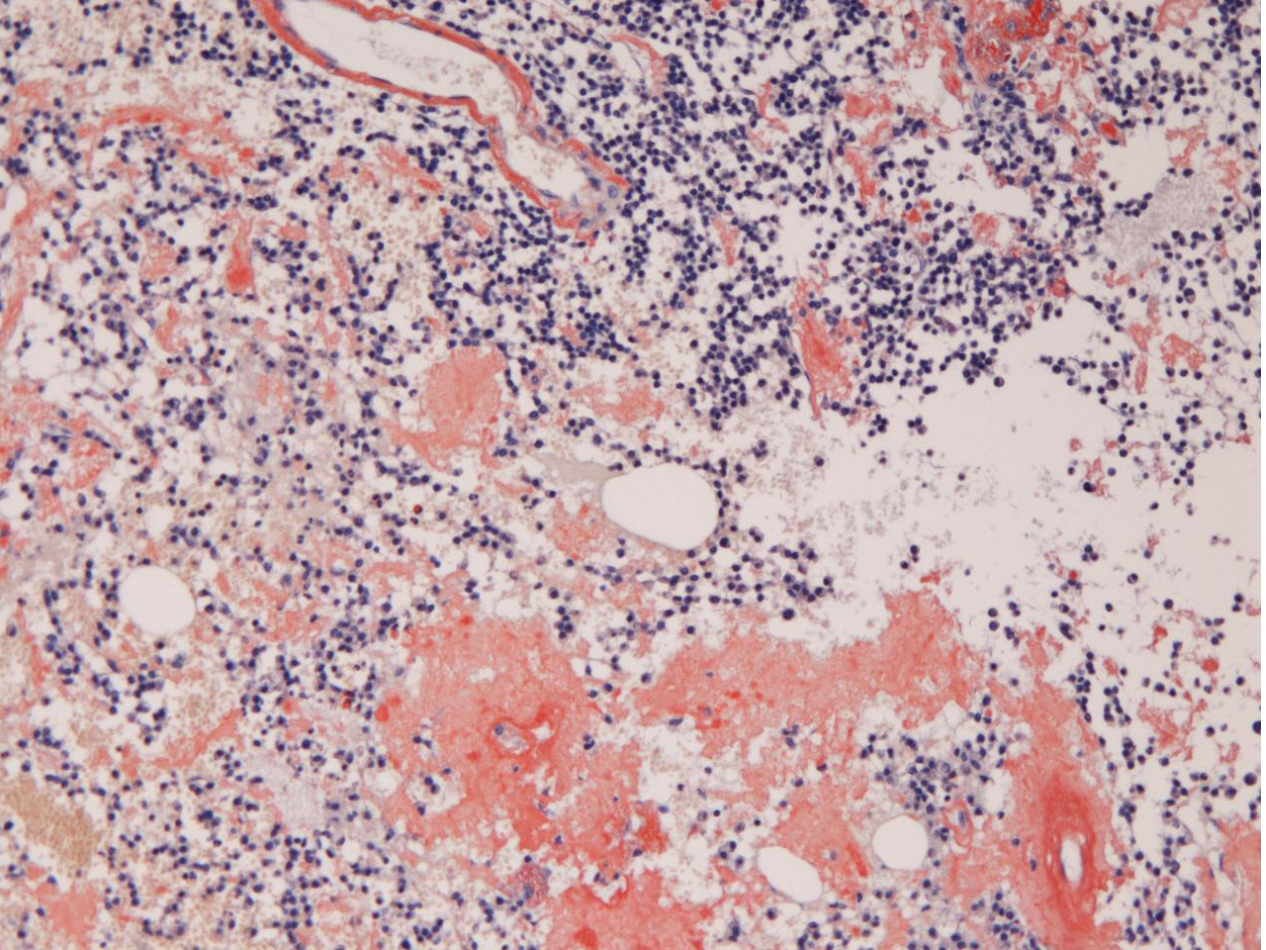
**Amyloid is scattered**. Congo-red stain, ×200.

Grossly, the brain tumor was soft, fragile, and bloody during the operation. Microscopically, a monotonous proliferation of atypical plasma cells with hyperchromatic nuclei was recognized (Figures 2 and 3). Histochemically, the tumor cells were pyroninophilic (Figure 4) and the congo-red stain revealed amyloidosis (Figure 5).

An immunohistochemical study was performed with the use of Dako Envision method (Dako, Glostrup, Denmark), as described previously [8,9]. The antibodies used were as follows: pancytokeratin (AE 1/3, Dako), pancytokeratin (CAM5.2, Beckton-Dickinson, CA, USA), epithelial membrane antigen (E29, Dako), CD45 (LCA, Dako), CD20 (L26, Dako), CD45RO (UCHL-1, Dako), κ-chain (polyclonal, Dako), λ-chain (polyclonal, Dako), IgG (polyclonal, Dako), IgM (polyclonal, Dako), IgA (polyclonal, Dako), CD68 (KP-1, Dako), synaptophysin (polyclonal Dako), chromogranin (DAK-A3, Dako), S100 protein (polyclonal, Dako), desmin (D33, Dako), α-smooth muscle antigen (1A4, Dako), myoglobin (polyclonal. Dako), p53 protein (DO7, Dako), Ki-67 antigen (MIB-I, Dako), and glial fibrillary acidic protein (GFAP) (6F2, Dako).

**Figure 6:**
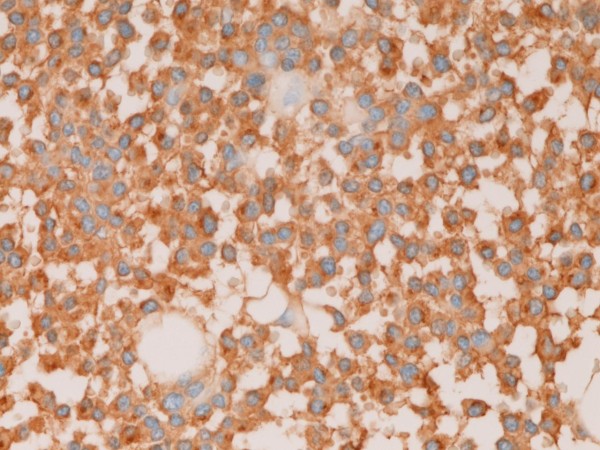
**The tumor cells are positive for κ light chain**. Immunostaining, ×400.

**Figure 7:**
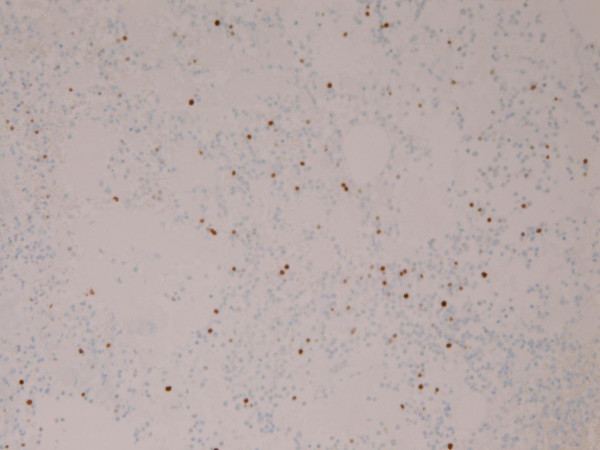
**The Ki-67 labeling is 11%**. Immunostaining, ×100.

The tumor cells were positive for κ-chain (Figure 6). However, they were negative for cytokeratin, epithelial membrane antigen, vimentin, CD45, CD20, CD45RO, λ-chain, IgM, IgA, IgG, synaptophysin, chromogranin, S100 protein, desmin, α-smooth muscle antigen, myoglobin, p53 protein, and GFAP. The Ki-67 labeling was 11% (Figure 7).

Intracranial plasmacytoma was pathologically diagnosed. The patient was treated by adjuvant chemoradiation, and entered into the complete remission stage. However, multiple metastases emerged in the vertebral bones and ribs six months after the remission. A diagnosis of MM was made. The urine revealed Bence-Jones protein of monoclonal IgG κ-chain type, but blood M protein was not recognized. The patient's condition gradually deteriorated. The patient died of respiratory failure due to bronchopneumonia 18 months after the admission. Autopsy was not performed.

## Discussion

MM or plasmacytoma presenting as an intracranial brain tumor is extremely rare. A review of the world literature revealed only 7 such cases. In the present case, the intracranial brain tumor was first diagnosed as chordoma, and signs and symptoms of MM including bone pain and blood M-protein were not recognized. The pathological examination, for the first time, revealed that the brain tumor was a plasmacytoma.

The pathologic diagnosis was apparently correct. Since, the plasma cells showed cellular atypia, the plasmacytoma was malignant. The pyroninophilia of tumor cells and the presence of amyloidosis strongly support the pathologic diagnosis of plasmacytoma. Further, the tumor cells were positive for κ-chain and negative for λ-chain, strongly suggesting the monoclonal proliferation of the tumor plasma cells. Curiously, the immunohistochemical study was negative for IgG, IgM, and IgA. Later, the urine test showed Bence-Jones protein with monoclonal IgG κ-chain. This may suggest that at first the tumor cells produced only κ light chain.

It is not certain whether the brain tumor was the manifestation of MM or the brain tumor of plasmacytoma gradually transformed into MM. Clinical course indicated that at first only the intracranial brain tumor was detected. After the adjuvant chemoradiation therapy, complete remission was obtained. The multiple bone lesions were detected six months after the operation and adjuvant chemoradiation therapy. These features may suggest that the intracranial plasmacytoma evolved into MM. However, there is a possibility that the intracranial brain tumor is the first manifestation of MM.

In summary, the present case indicates that MM may be preceded by or manifested as an intracranial brain tumor (plasmacytoma).

## Consent

Written informed consent was obtained from the patient for publication of this case report and accompanying images. A copy of the written consent is available for review by the Editor-in-Chief of this journal.

## Competing interests

The author declares that they have no competing interests.
